# Numerical and Experimental Analysis of the Rotational Stiffness of a Timber Semi-Rigid Dowel-Type Connection

**DOI:** 10.3390/ma15165622

**Published:** 2022-08-16

**Authors:** Marek Johanides, Antonin Lokaj, Pavel Dobes, David Mikolasek

**Affiliations:** 1 Department of Structures, Faculty of Civil Engineering, VSB-Technical University of Ostrava, 708 00 Ostrava, Czech Republic; 2 Centre for Building Experiments and Diagnostics, Faculty of Civil Engineering, VSB-Technical University of Ostrava, 708 00 Ostrava, Czech Republic

**Keywords:** bolts, dowels, dowel-type fasteners, FEM, frame connection, fully threaded screws, glued laminated timber, numerical model, rotational stiffness

## Abstract

The paper deals with the analysis of the rotational stiffness of a semirigid connection created from a system of two stands and a rung. The connection was made from glued laminated timber with metal mechanical dowel-type fasteners. Not only a common combination of bolts and dowels but also fully threaded screws were used for the connection. The aim of the research and its motivation was to replace commonly used fasteners with more modern ones, to shorten and simplify the assembly time, and to improve the load-carrying capacity of this type of connection. Each of these two types of connection was loaded to the level of 60%, 80%, and 100% of the ultimate limit state value. Subsequently, the rotational stiffness was determined for each load level after five loading and unloading cycles. This paper presents the results and comparison of the experimental testing and the numerical modeling. The obtained results were also compared with the assumption according to the currently valid standard.

## 1. Introduction

Stiffness is an important property of connections in timber structures. When designing and assessing a structure, it is necessary to take into account not only the load-carrying capacity but also the deformation behavior in order to ensure sufficient reliability of the structure. In 1949, Granholm [1] began to deal with the issue of deformation capacity of structures after the fall of a formwork of the Sandö Bridge, which was located in Sweden in the 1930s. The failure of the structure was caused by ignoring the nonlinear deformation behavior of fasteners in connections. The scientist found that the stiffness was not constant and varied depending on the load level and deformation of the connection. A decrease in connection stiffness can be observed with increasing deformation. In 1963, Granholm [2] determined the tangent and secant stiffness along the entire curve of a load–deformation diagram for a nail connection. The stiffness was calculated based on 70 load–deformation curves for a nail diameter of 5.6 mm and a length of 150 mm. The scientist also emphasized consideration the friction between the timber elements in the connection. In the case of the collapse of the aforementioned bridge formwork, the stiffness at failure was only about 20–25% of the initial values.

Ehlbeck [3] identified some parameters that influence the load–deformation behavior. These parameters are divided into four main parts. The dimensions and material properties of fasteners represent the first part. These properties include the size, length, diameter, surface treatment, and bending properties of the fastener. The second part includes mechanical properties of fasteners, such as slip moduli and yield moment. The third part contains configurations of fasteners, i.e., number of shear planes, thickness of timber element, predrilling of holes, and spacing and distances for fasteners. The last part of influences includes loading conditions. These can be static or dynamic, short term or long term, and also loading rate. The identification of these parameters means that the stiffness values given in the design codes are only rough estimates, due to the great variability of material properties [3].

In 1981, Dubas and Gehri [4] stated that the standard at the time took into account only the slip modulus corresponding to the ultimate limit state. This failure stiffness was too conservative for the serviceability limit state due to the strong dependence of the respective stiffness value on the respective load value. It was also emphasized that the initial slip of the connection is usually smaller for tested connections than for actual connections made on the construction site in practice. This is due to greater accuracy and precision under laboratory conditions. They also stated that the initial slip can be observed for all types of connections, even for predrilled ones. It is possible to expect an initial slip of 0.50–1.00 mm depending on the fastener diameter or timber drying. There are very few experiments and scientific work that have evaluated the effect of moisture content on connection stiffness 2020 [5]. That latter work assumes that the most deformations in the connection occur in the timber element, so the same moisture content dependence can be applied to the connection stiffness. Furthermore, the paper states that as the moisture content of the timber element increases, its modulus of elasticity decreases and thus the deformation increases. However, this idea needs to be verified by several studies.

In experimental tests by Ehlbeck and Werner in 1988 [6], it was observed that deformation under the allowable load according to DIN 1052 [7] increased with increasing fastener diameter. They derived an equation for calculation of the slip modulus. This was later modified by Ehlbeck and Larsen [8], and became the standard equation for the calculation of the slip modulus. This relationship was and is still under investigation. Jorissen [9] also dealt with this relation in his dissertation and found that in the case of multiple fastener connections, the stiffness values were significantly lower than assumed by the current standard approach. This is explained by the clearances for holes and the nonuniform load distribution of individual fasteners during loading.

Nowadays, the slip modulus for fasteners (translational stiffness) is calculated on the basis of the equations given in Eurocode 5 [10]. However, this standard does not specify a procedure for calculating rotational stiffness. Rotational stiffness can be calculated on the basis of the equations given in the scientific literature [11] using the slip modulus according to Eurocode 5 [9].

Dowel-type fasteners, such as screws, bolts, and dowels, are one of the most popular fasteners in timber structures [12,13]. They are easy to use, cheap, and available everywhere. In order to effectively apply dowel-type connections, it is crucial to understand their mechanical behavior under loading. It is desirable to know the relationship between load and slip, stress distribution, or possible different failure modes. The mechanical behavior of connections in timber structures is a complex problem that is influenced by a number of factors [14]. The most important factors are the geometry and arrangement of the connection (i.e., spacing, edge and end distances) [15], the material characteristics, the type of timber [16], and the method of loading.

The load-carrying capacity and stiffness of connections are influenced not only by mechanical properties of fasteners but also by mechanical properties of timber. The stiffness is mainly affected by the timber density. The findings described in [17,18] can be used to determine the mechanical properties and to classify the structural timber. It is possible to use selected nondestructive experimental testing [19,20], dynamic testing [21], and a nondestructive vibrational method [22] in order to determine these mechanical properties. A semidestructive method can also be used to determine the density and moisture content of timber [23].

Nowadays, numerical modeling should also be included as an important part of experiments. Numerical modeling is an excellent tool to understand the behavior of connections in timber structures. Dobeš [24], Braun [25], and Kupniewska [26] dealt with the calibration and validation of numerical models according to experimental tests. The papers stated that numerical modeling was especially suitable for determining the stress in individual elements of the connection and determining the locations of stress peaks. However, the exact prediction of the load–deformation response is often problematic, because the response of the tested connection is influenced by many factors that can hardly be taken into account in the numerical model (e.g., unpredictable initial slip and the gradual increase in stiffness in the initial consolidation phase of the connection when the contact between the timber and the fastener is just forming).

The paper is focused on the experimental determination of the rotational stiffness of a semirigid connection, with subsequent validation of a numerical model. The fist variant of the connection is made of a combination of bolts and dowels in Experiment A. The second variant of the connection is made of high tensile fully threaded screws in Experiment B. The fasteners in Experiment A are used in practice, so it is a common combination. Knowledge on the design of such a semirigid connection can be found elsewhere [27,28,29]. However, the fasteners in Experiment B are not commonly used in practice. The presented paper is a follow-up of previous research [30,31,32]. Papers [30,31] dealt with determining the rotational stiffness and load-carrying capacity of a timber frame corner. However, this structure was much larger and made of larger cross-sections with greater number of fasteners. The aim was to create a connection with higher load-carrying capacity using the same structural elements. Johanides [32] dealt with smaller timber frame corners. The paper determined and compared the ductility of these two types of connection (bolts and dowels vs. fully threaded screws).

This paper brings knowledge about the results of analytical, experimental, and numerical determination of the rotational stiffness of a semirigid connection with mechanical fasteners. The reader can view the results of a comparison of two types of fasteners and form his/her own opinion on the advantages and disadvantages of individual fasteners based on the presented data.

## 2. Materials and Methods

### 2.1. Description of Structure and Geometry

The tested specimens corresponded to actual connections used in practice. The arrangement and loading of test specimens was designed to correspond with the actual state of the connection in a real load-carrying structure. The air temperature was 21 °C and the relative air humidity 55% during the experimental testing.

The structural system for the experiments was created from a semirigid connection of two stands and a rung. These structural elements were made of spruce timber, which is the most used structural timber in Central Europe for structural practice due to its availability. The disadvantage of this timber is its low durability in the outdoor environment. The connection was made of dowel-type metal fasteners. Two identical structural systems were created with different types of fasteners. Experiment A contained a combination of bolts and dowels as fasteners. Experiment B contained fully threaded screws. Glued laminated timber of the strength class GL24h was used. Four-point bending tests were performed to verify the properties of the used timber. Results of the tests have already been published [32]. The stands were made of a cross-section of 100/300 mm and the rung was made of a cross-section of 100/300 mm. The material properties of the fasteners were also experimentally verified by tensile tests [32]. Bolts and dowels in Experiment A were made of steel, grade 10.9. The outer diameter of the threaded shank was 8 mm, the inner diameter was 7.25 mm. The bolt length was 360 mm and the dowel length was 300 mm. Holes for fasteners were predrilled with an 8 mm-diameter drill to get connections without initial slip. The fully threaded bolts in Experiment B were made of steel, grade 10.9. The outer diameter of the screw was 8 mm, the inner diameter of the screw was 5 mm, and the length was 300 mm. Holes for fasteners were predrilled with a 5 mm-diameter drill, also in order to eliminate the initial slip.

**Figure 1:**
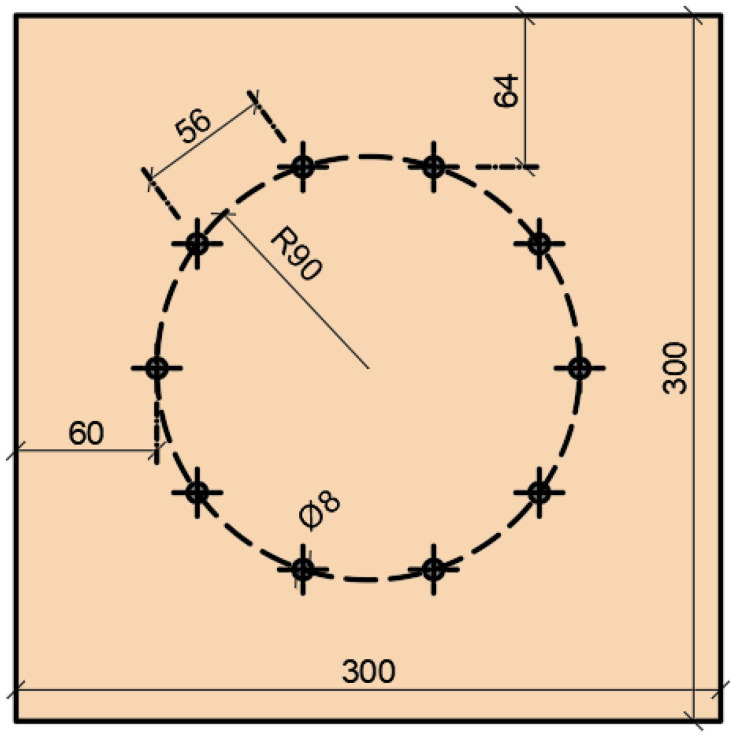
The arrangement of fasteners.

The arrangement of fasteners in both experiments was identical. They were located on one symmetrical circle with a radius *r* = 90 mm, with 10 pieces. The arrangement (see Figure 1) was determined according to [11].

**Figure 2:**
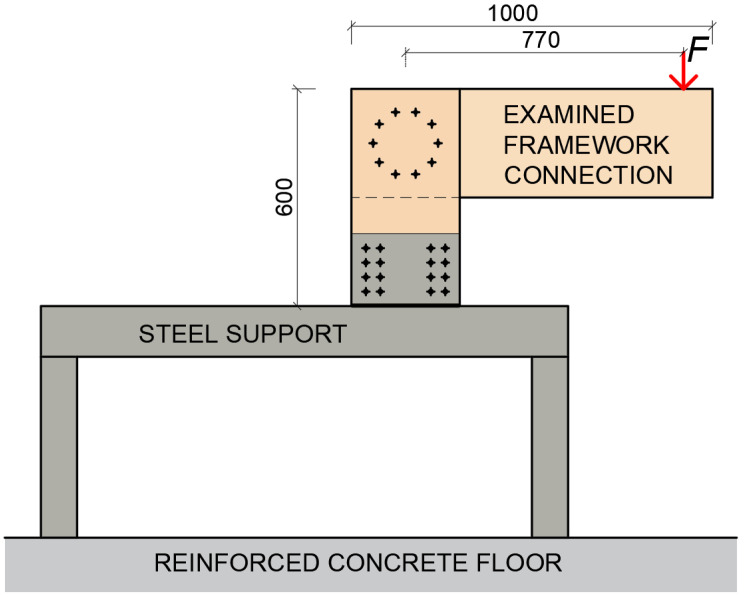
Schematic illustration of the experiment.

It was necessary to create the boundary conditions of the structure to carry out these experiments. A steel structure was designed and made to ensure the correct boundary conditions. A steel sheet of the desired shape was fastened to the steel structure by bolts and subsequently the stand of the semirigid connection was fastened to the plate using 24 bolts with a diameter of 8 mm. This connection was calculated and designed for 400% of the estimated load that would cause the connection failure. The entire steel structure was placed on a reinforced concrete floor at the required distance from the testing machine so the load could be applied to the required position on the rung. After the correct positioning of the steel structure, the rear part of the steel structure was loaded with a steel box with a weight of 1000 kg against its overturning and displacement. A schematic illustration of the experiment is shown in Figure 2.

### 2.2. Description of the Testing Machine

The experiments were performed using a LabTest 6.1200 electromechanical testing machine from Labortech (Opava, Czech Republic) [33] with a maximum force of 1200 kN. This testing machine allows tensile, compressive static, and cyclic dynamic testing. The test speed of the testing machine ranges from 0.0005 to 250 mm/min. The machine and testing procedure is controlled by computer software.

### 2.3. Position of Gauge Sensors

**Figure 3:**
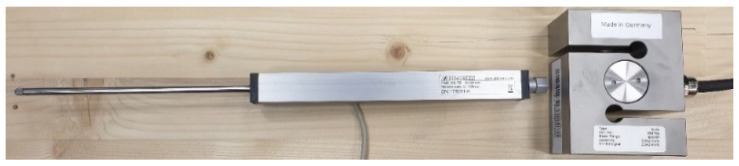
Strain gauge sensor (on the left); force gauge sensor (on the right).

**Figure 4:**
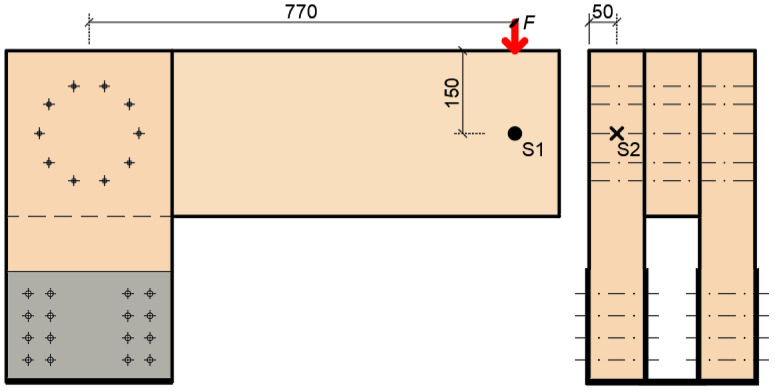
Position of strain gauge sensors: front view (on the left), side view (on the right).

To evaluate the rotational stiffness from the tests, it was necessary to obtain the most accurate input data for the calculation. This was achieved by using the force gauge sensors (see Figure 3 on the right), which was located under the crosshead. The ALMEMO FKA0255 gauge sensor records force up to 50 kN with an accuracy of ±0.20% in pressure and ±0.10% tension. Furthermore, it was necessary to fit the strain gauge sensors correctly (see Figure 3 on the left), according to the scheme in Figure 4. An Almemo FWA100TR gauge sensor with a range of 0–100 mm with an accuracy of ±0.002 mm was used to record the deformation. Both sensors were from Ahlborn (Holzkirchen, Germany) [34].

### 2.4. Determination of the Rotational Stiffness

The system for loading (see Figure 2) included several influences that cause the vertical movement of the rung end. These components must be subtracted to determine the actual deformation caused by the rotational stiffness of the frame connection. The following text therefore focuses on determination of the deformation caused by the rotational stiffness.

**Figure 5:**
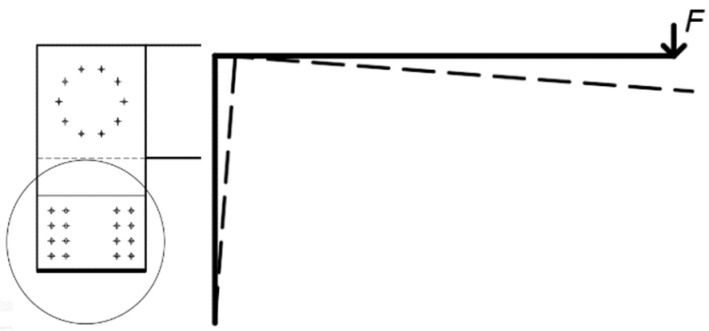
Semirigid connection between the stand and the steel plate (on the left); deformation due to inclination (on the right).

The first important factor is the semirigid connection between the lower part of the stand and the supporting steel structure (see Figure 5). This connection causes an inclination of the stand during loading, and subsequently a vertical displacement of the rung end. Therefore, one strain gauge sensor is placed horizontally (S2 in Figure 4). It records the horizontal displacement (inclination) of the stand. It is then possible to determine this vertical deformation based on the obtained data.

**Figure 6:**
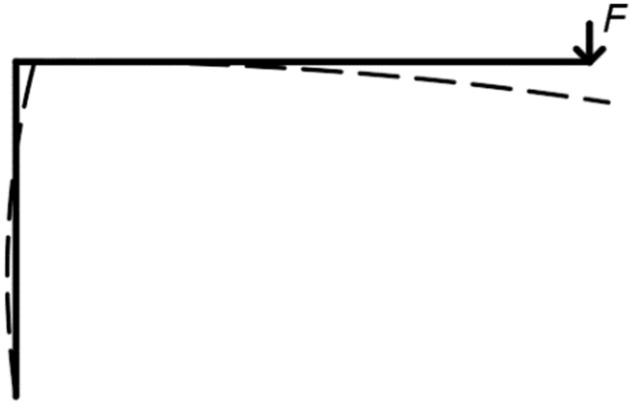
Deformation of segments due to bending moment and shear force.

It is also necessary to take into account deformations of the individual segments (see Figure 6), caused by the bending moment and the shear force. These deformations can be determined analytically according to cross-sectional characteristics and dimensions of the individual segments, for example, by means of the force method.

**Figure 7:**
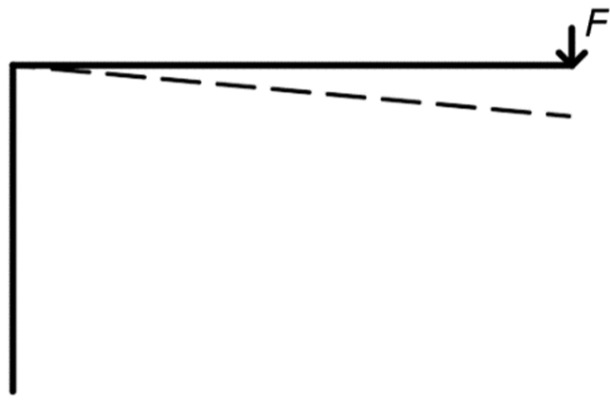
Deformation of the rung end caused by the rotational stiffness of the frame connection.

The actual value of the deformation caused by the rotational stiffness can be obtained if the abovementioned effects of the vertical displacements are subtracted from the total displacement measured by the sensor S1 (see Figure 4). Based on the obtained value of deformation, it is possible to determine the course of rotational stiffness depending on the applied load (see Figure 7). It can subsequently be compared with the value from the numerical model and analytical calculation [11].

### 2.5. Description of the Loading Procedure of Quasi-Static Tests

The aim of the quasi-static cyclic testing was to investigate the behavior of the semirigid connection at different load levels. The rotational stiffness values for the individual load levels were determined based on the obtained data.

The idea of such testing is built on a practical basis. This means that building structures are designed with consideration of the ultimate limit state. The limit value of the load-carrying capacity must not be exceeded in a real structure; otherwise, there is a risk of permanent damage of the supporting structure, or its collapse [10]. For this reason, the rotational stiffness of the connection beyond the ultimate limit state was not investigated. The selection of individual load levels for the cyclic testing was chosen strategically as follows.

1. The first value for loading was calculated as 60% of the ultimate limit state value of load-carrying capacity. This load represents a common design situation of most load-carrying structures.
2. The second value for loading was calculated as 80% of the ultimate limit state value of load-carrying capacity. This load is also a common design situation of some load-carrying structures.
3. The third value for loading was calculated as 100% of the ultimate limit state value of load-carrying capacity. This load represents the maximum load of load-carrying structures in practice.

The tensile load was generated by electrohydraulic cylinders. During the test, the time, tensile force, and deformation (i.e., crosshead displacement) were continuously recorded. Strain gauge sensors were installed on the structures in order to enable a correct evaluation of the results (see Section 2.3).

The test specimens were subjected to nondestructive tests before the cyclic testing to determine the moisture content and density of the used timber. The timber density is the main factor in the analytical calculation of the slip modulus of fasteners according to Eurocode 5 [10] and thus also the rotational stiffness according to Koželouh [11].

It is important to note that a new unloaded connection was used for each test to achieve correct results. Using an already loaded specimen would affect the results, as a consequence of embedment of timber or irreversible permanent deformation of fasteners.

The following loading procedure is a modified approach of the authors, which is based on the standard EN 26891 [35].

1. Calculation of the maximum force *F_ed_* for the tested connection (i.e., 100% ULS);
2. Loading of the specimen to 60% *F_ed_*, then holding for 30 s;
3. Unloading to 10% *F_ed_*, then holding for 30 s;
4. Repeating steps 2 and 3 four times, until a total of 5 load cycles is done.

The procedure mentioned in the text above was also applied to test specimens at 80% and 100% *F_ed_*.

Table 1 shows the values that determine the course of the experimental loading for Experiment A (bolts and dowels) and Experiment B (fully threaded screws) at 60% of the ultimate limit state value. The load-carrying capacity of the connection *F_ed_* was calculated as the design value (using modification factor *k_mod_* = 0.90 and partial factor for material properties *γ_con_* = 1.30). This value represents the maximum load-carrying capacity of the connection and was calculated according to Eurocode 5 [10] and the literature [10].

The loading speed was chosen as constant in kN/min according to the selected loading schemes. The total testing time of one specimen was 30 min.

**Figure 8:**
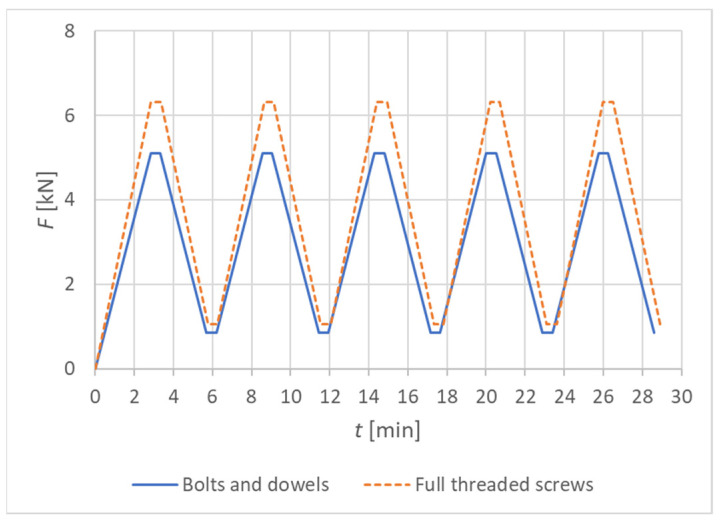
The course of individual experiments.

Figure 8 shows the graphic course of individual experimental tests.

### 2.6. Experimental Testing

**Figure 9:**
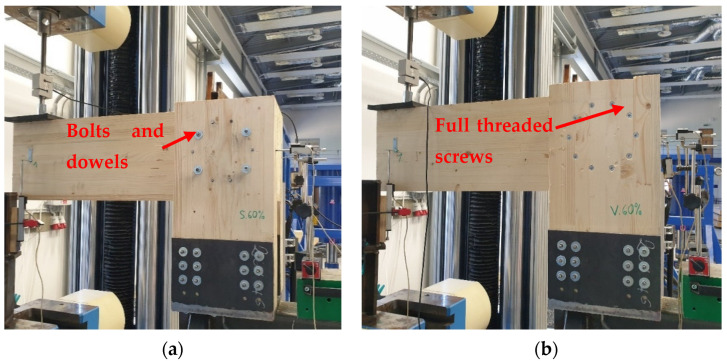
Experimental specimens: (**a**) Experiment A, bolts and dowels; (**b**) Experiment B, fully threaded screws.

All the experiments were carried out at the Centre for Building Experiments and Diagnostics at VSB—Technical University of Ostrava, Czech Republic. Figure 9a shows Experiment A, a combination of bolts and dowels. Figure 9b shows Experiment B, fully threaded screws. The load was applied to the connection using a steel cylinder with a diameter of 50 mm. A rubber pad with a thickness of 10 mm was placed under this cylinder to eliminate local damage of the timber rung during loading.

### 2.7. Numerical Modeling

The numerical model was created in the Ansys 21 software in the Workbench 21 environment (Canonsburg, PA, USA) [36]. The models used 3D finite elements with the support of material nonlinearity, geometric nonlinearity, and contact elements. The material model of timber was considered to be orthotropic with the Hill yield criterion to predict plastic behavior, [37]. Steel elements were considered isotropic with plastic behavior based on the Von Mises yield criterion [37]. The load–deformation response of the numerical model would not be sufficiently accurate without considering the orthotropy of timber and the plastic behavior of materials. Mikolášek [38] and Gunderson and Goodman [39] were also used to gain the material characteristics of timber for the numerical models. The material characteristics of timber are shown in Table 2, and the material characteristics of steel are shown in Table 3.

The modulus of elasticity of fasteners was determined on the basis of the experience of the authors.

The values of the plastic behavior of timber (see Table 4), and the plastic behavior of steel (see Table 5) were obtained based on an experimental testing. The results of the testing have already been published in [31].

**Figure 10:**
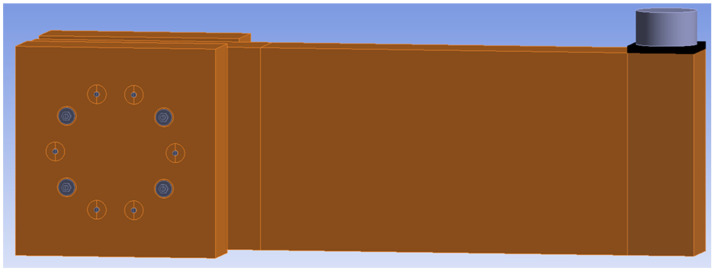
Numerical model for Experiment A, bolts and dowels.

**Figure 11:**
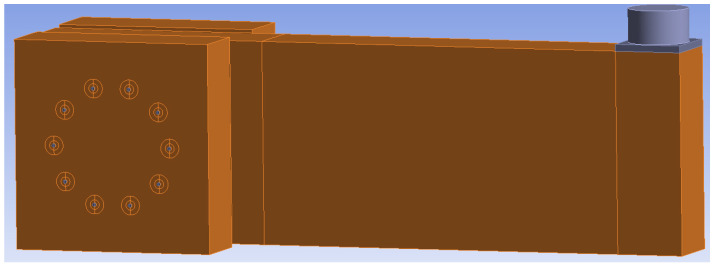
Numerical model for Experiment B, fully threaded screws.

Figure 10 and Figure 11 show individual numerical models that were used for the analysis of the rotational stiffness at different load levels after five cycles of loading and unloading. The connection with bolts and dowels (Experiment A) contained 125,000 nodes, 32,467 finite elements and 375,000 equations. The connection with fully threaded screws (Experiment B) contained 78,557 nodes, 15,868 elements, and 218,729 equations.

**Figure 12:**
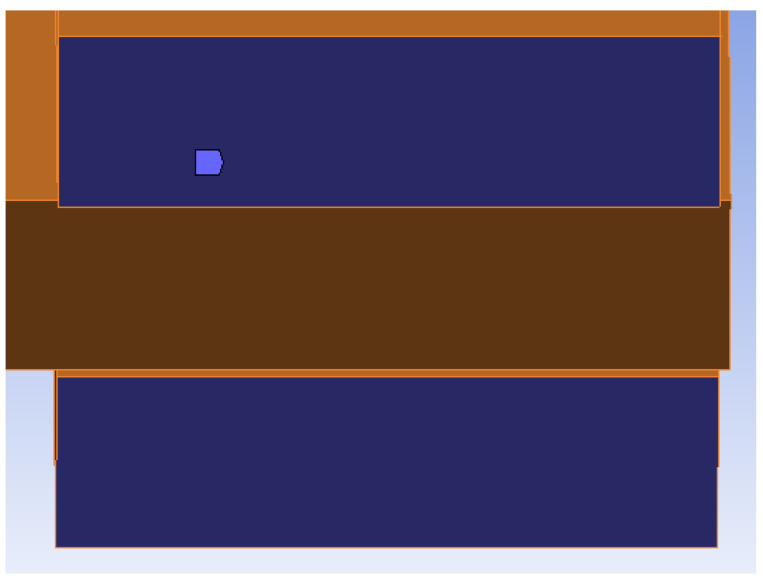
Boundary conditions of the numerical models.

Thanks to carefully placed strain gauge sensors and analytical calculations described in Section 2.3, it was possible to choose a fixed support for the stands as boundary conditions. After subtracting the already mentioned external influences causing the deformation of the rung, we can get the deformation caused by the rotational stiffness of the connection. Figure 12 shows the selected boundary conditions of the numerical model (the fixed support of the stands).

**Figure 13:**
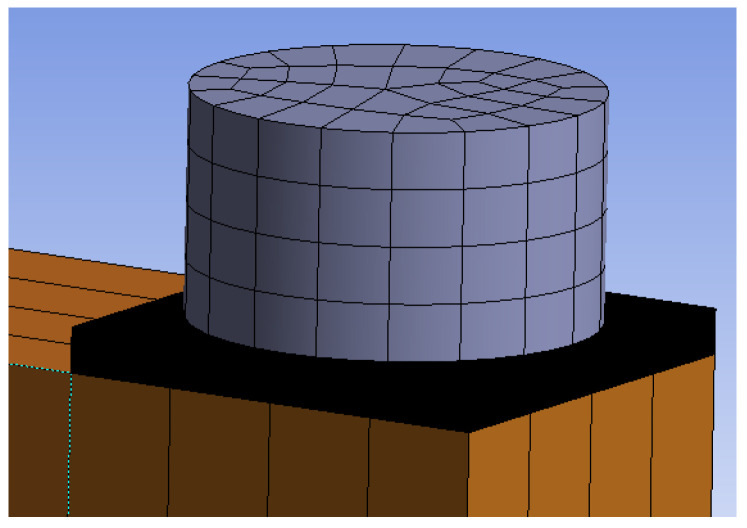
Finite-element mesh for applying load.

It is very important to correctly apply the load into the numerical model. Figure 13 shows the finite-element mesh for both numerical models for applying the load using the press head (cylinder) of the testing machine. The model was loaded by a vertical displacement (displacement controlled loading), which represented the actual loading by the cylinder during the experimental testing.

**Figure 14:**
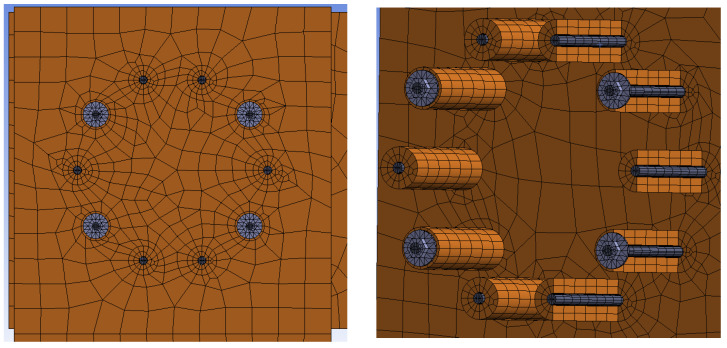
Finite-element mesh for Experiment A, bolts and dowels.

**Figure 15:**
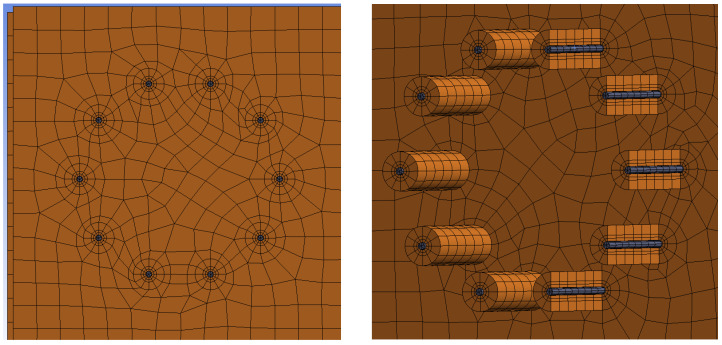
Finite-element mesh for Experiment B, fully threaded screws.

The mesh was created from 3D hexahedral finite elements. The finite-element mesh was divided into several subregions with different finite-element sizes with respect to the estimated areas of increased local stress concentrations. A finer mesh was chosen around the contact of the fastener with the timber element. A ratio of 3:1 (length to height) for the finite elements of the subregions was set to obtain optimal results. The interface between individual elements was simulated using frictional contacts. The coefficient of friction between timber–timber elements was 0.40, steel–steel 0.10, and timber–steel 0.30. Figure 14 shows the finite-element mesh for Experiment A (bolts and dowels). Figure 15 shows the finite-element mesh for Experiment B (fully threaded screws).

## 3. Results

### 3.1. Results of Experimental Testing

#### 3.1.1. Experiment A, Bolts and Dowels

**Figure 16:**
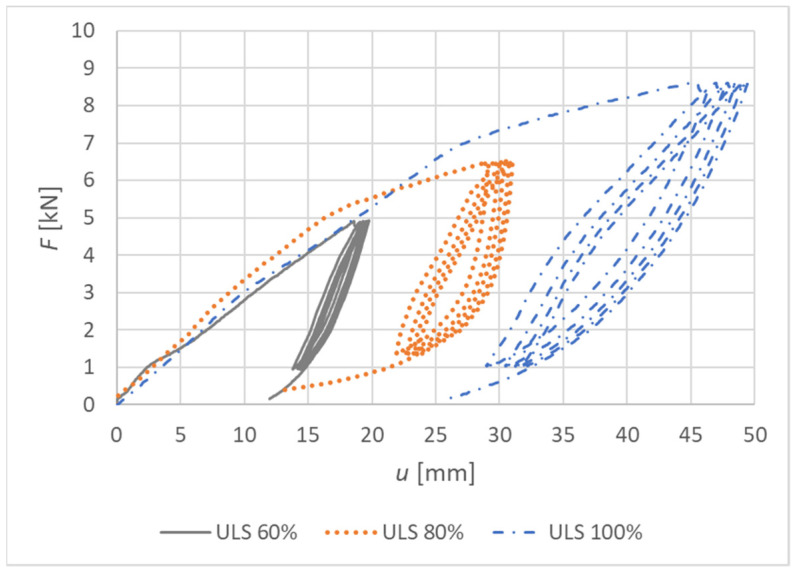
Load–deformation curves of nondestructive quasi-static cyclic testing for individual specimens made from a combination of bolts and dowels (Experiment A).

Figure 16 shows load–deformation curves from the experimental testing. The deformation *u* represents the actual vertical deformation of the rung end after subtracting all the influences that were explained in Section 2.3. The loading procedure was carried out according to the explanation in Section 2.4, and the position of the load is shown in Figure 4.

**Figure 17:**
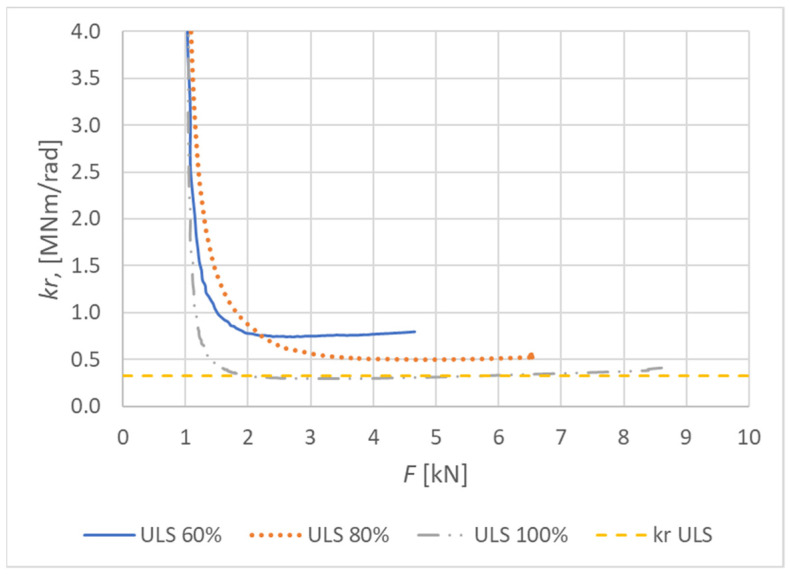
Course of rotational stiffness of a semirigid connection for specimens made from a combination of bolts and dowels (Experiment A).

Figure 17 shows the curves of the rotational stiffness depending on the load level for the connection made from a combination of bolts and dowels. The figure also indicates the rotational stiffness values calculated for the ultimate limit state according to Eurocode 5 [10].

The results of the individual tests are listed in Table 6, Table 7 and Table 8. The first column indicates the load level. The second column shows the maximum force achieved during the test. The third column shows the corresponding value of the bending moment. The fourth column shows the actual deformation caused by the rotational stiffness of the semirigid connection. The fifth column contains the calculated value of the rotational stiffness based on the experimental data. The sixth and the seventh columns contain the standard deviation value and the average value of the experimental data. The eighth column contains the calculated rotational stiffness according to Eurocode 5 [10]. The ninth column shows the ratio between the rotational stiffness obtained experimentally and the rotational stiffness calculated according to Eurocode 5 [10].

**Figure 18:**
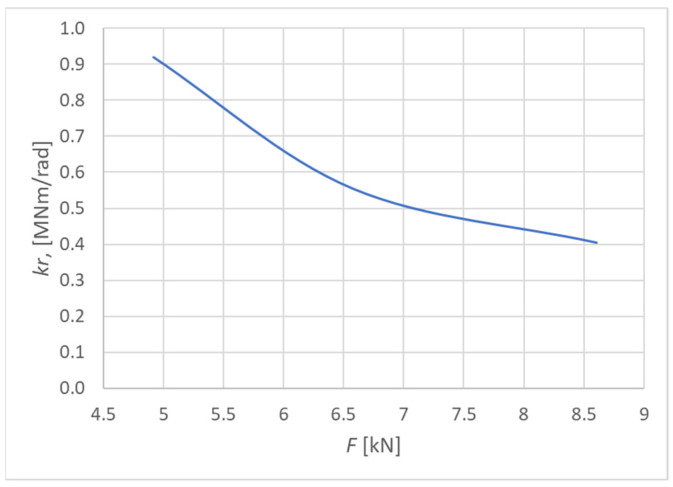
Trend of rotational stiffness for Experiment A.

Figure 18 shows the course of the rotational stiffness of the connection. It was created on the basis of the individual load levels. It is possible to observe a decreasing trend in the level of rotational stiffness with the increasing load.

#### 3.1.2. Experiment B, Fully Threaded Screws

**Figure 19:**
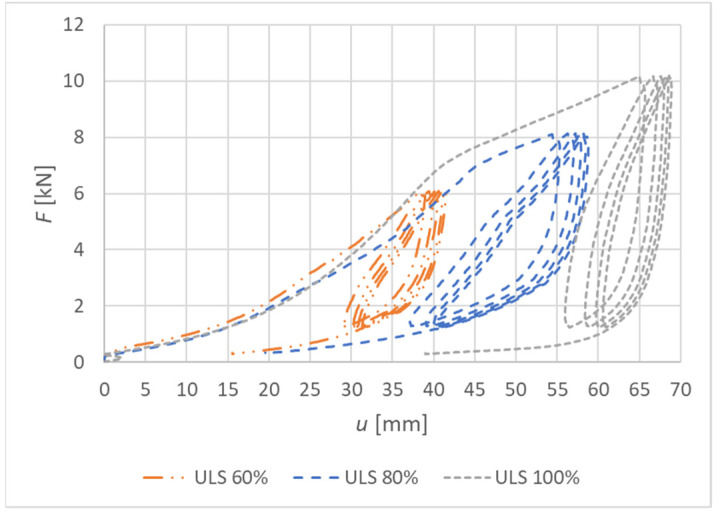
Load–deformation curves of nondestructive quasi-static cyclic testing for individual specimens made from fully threaded screws (Experiment B).

Figure 19 shows load–deformation curves from the experimental testing. The deformation *u* represents the actual vertical deformation of the rung end after subtracting all the influences that were explained in Section 2.3. The loading procedure were carried out according to the explanation in Section 2.4, and the position of the load is shown in Figure 4.

**Figure 20:**
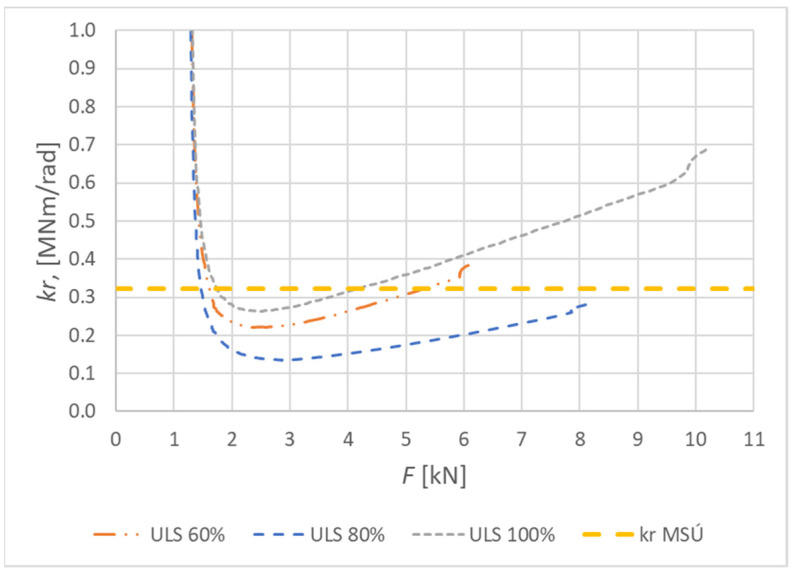
Course of rotational stiffness of a semirigid connection for specimens made from fully threaded screws (Experiment B).

Figure 20 shows the curves of the rotational stiffness depending on the load level for the connection made from fully threaded screws. The figure also indicates the rotational stiffness values calculated for the ultimate limit state according to Eurocode 5 [10].

The results of the individual tests are listed in Table 9, Table 10 and Table 11. The first column indicates the load level. The second column shows the maximum force achieved during loading. The third column shows the corresponding value of the bending moment. The fourth column shows the actual deformation caused by the rotational stiffness of the semirigid connection. The fifth column contains the calculated value of the rotational stiffness based on the experimental data. The sixth and the seventh columns contain the standard deviation value and the average value of the experimental data. The eighth column contains the calculated rotational stiffness according to Eurocode 5 [10]. The ninth column shows the ratio between the rotational stiffness obtained experimentally and the rotational stiffness calculated according to Eurocode 5 [10].

**Figure 21:**
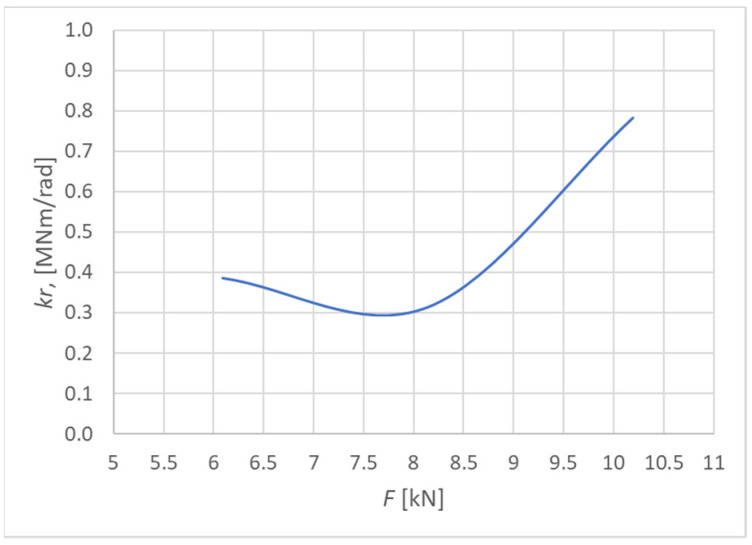
Trend of rotational stiffness for Experiment B.

Figure 21 shows the course of the rotational stiffness of the connection. It was created on the basis of the individual load levels. It is possible to observe an increase in the rotational stiffness with the increasing load.

### 3.2. Results of the Numerical Modeling

#### 3.2.1. Experiment A, Bolts and Dowels

**Figure 22:**
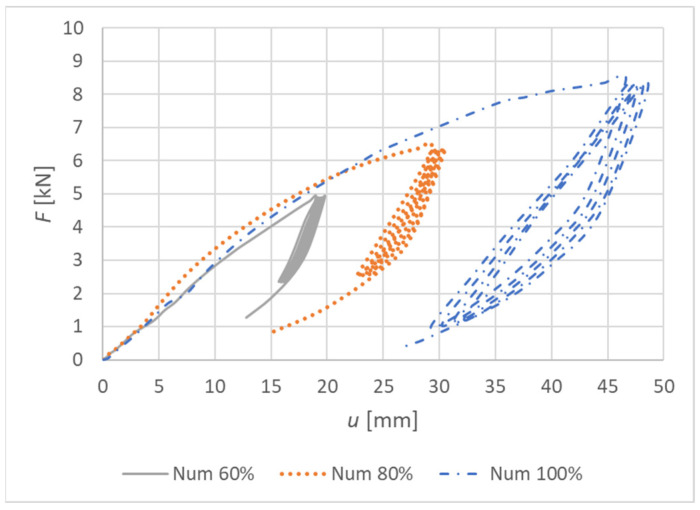
Load–deformation curves of quasi-static numerical modeling for individual specimens made from a combination of bolts and dowels (Experiment A).

Figure 22 shows load–deformation curves from the numerical models. The deformation *u* represents the vertical deformation of the rung end in the numerical model.

**Figure 23:**
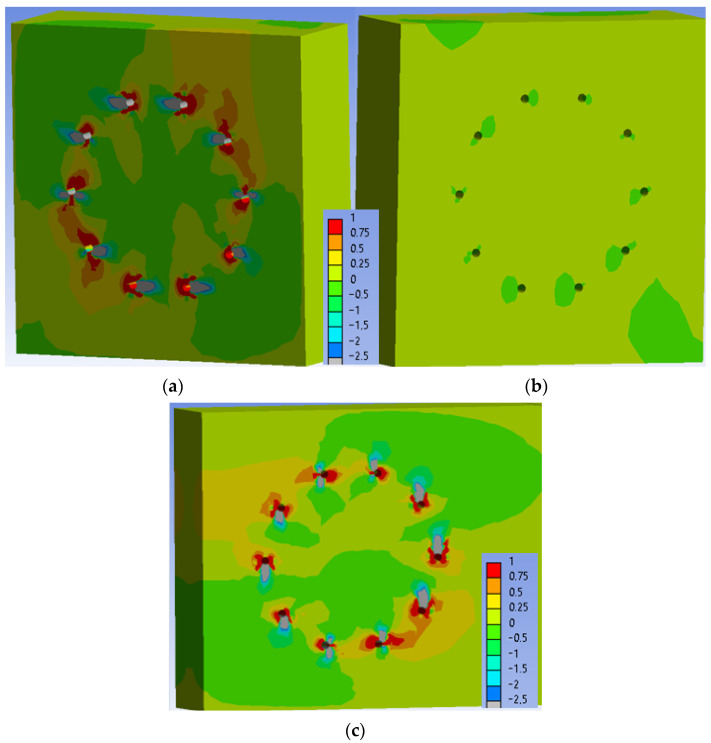
Tensile stress perpendicular to the grain [MPa] at 60% ULS, Experiment A: (**a**) inner side of the stand; (**b**) outer side of the stand; (**c**) rung.

Figure 23 shows the normal stress perpendicular to the grain in the individual elements of the connection at a load level of 60% ULS. As can be seen, the maximum tensile stress perpendicular to the grain (1 MPa) is locally concentrated mainly near the holes.

**Figure 24:**
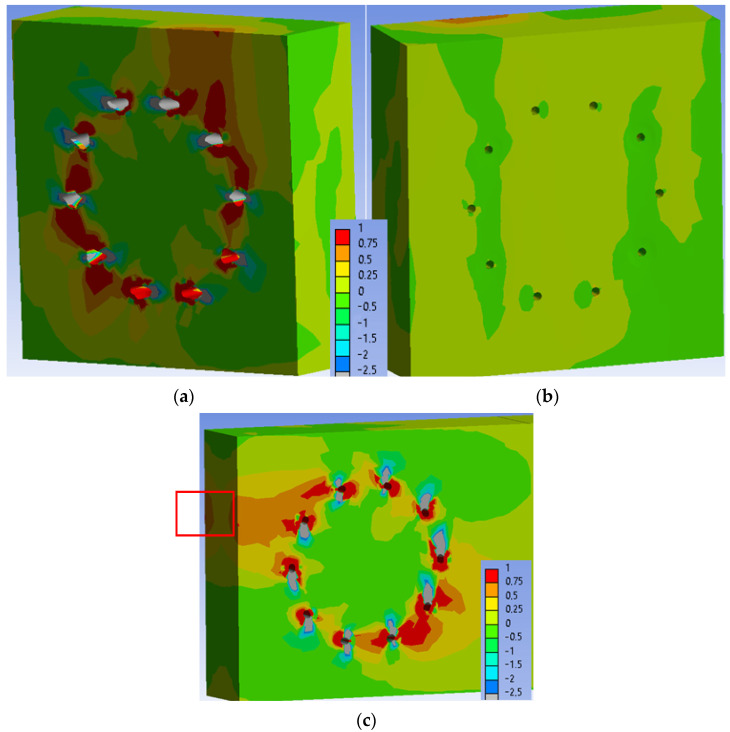
Tensile stress perpendicular to the grain [MPa] at 80% ULS, Experiment A: (**a**) inner side of the stand; (**b**) outer side of the stand; (**c**) rung.

Figure 24 shows the normal stress perpendicular to the grain in the individual elements of the connection at a load level of 80% ULS. The maximum tensile stress perpendicular to the grain (1 MPa) is also concentrated mainly near the holes at this load level. However, the stress in the critical area of the connection (marked in Figure 24c) increased to values of 0.25–0.50 MPa. This stress did not cause any visible cracks in the timber elements during the testing.

**Figure 25:**
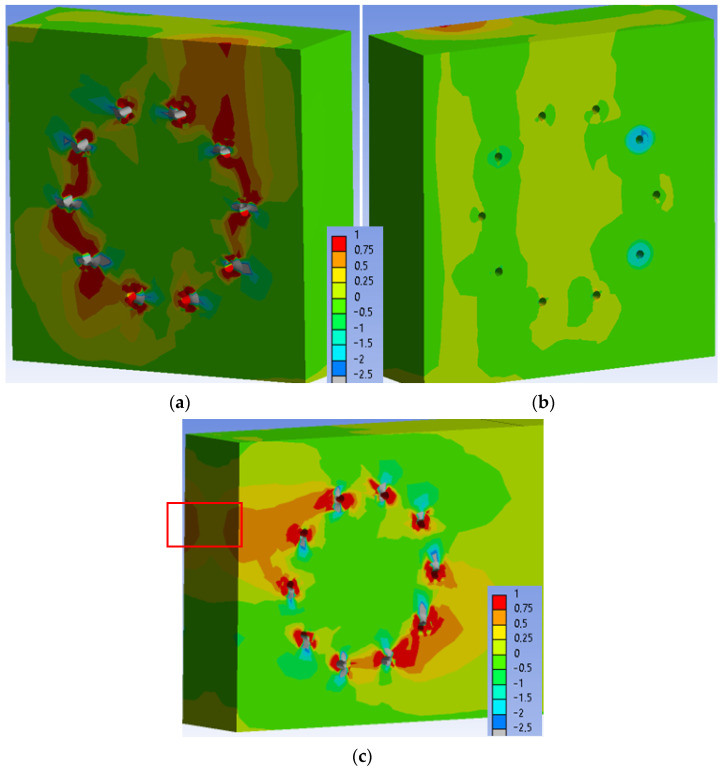
Tensile stress perpendicular to the grain [MPa] at 100% ULS, Experiment A: (**a**) inner side of the stand; (**b**) outer side of the stand; (**c**) rung.

Figure 25 shows the normal stress perpendicular to the grain in the individual elements of the connection at a load level of 100% ULS. Areas of the maximum tensile stress perpendicular to the grain (1 MPa) continue to expand around the holes at this load level. The stress in the critical area of the connection (marked in Figure 25c) remained unchanged with values of 0.25–0.50 MPa. This stress did not cause any visible cracks in the timber elements during the testing.

The results of the individual numerical models are listed in Table 12, Table 13 and Table 14. The first column indicates the load level. The second column shows the maximum force achieved during loading. The third column shows the corresponding value of the bending moment. The fourth column shows the actual deformation caused by the rotational stiffness of the semirigid connection. The fifth column contains the calculated value of the rotational stiffness based on the numerical model. The sixth and the seventh columns contain the standard deviation value and the average value of the experimental data. The eighth column contains the calculated rotational stiffness according to Eurocode 5 [10]. The ninth column shows the ratio between the rotational stiffness obtained from the numerical analysis and the rotational stiffness calculated according to Eurocode 5 [10].

#### 3.2.2. Experiment B, Fully Threaded Screws

**Figure 26:**
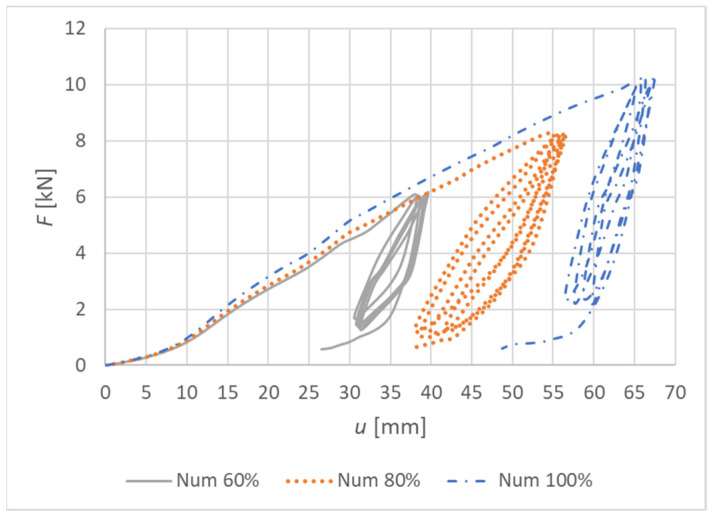
Load–deformation curves of quasi-static numerical modeling for individual specimens made from fully threaded screws (Experiment B).

Figure 26 shows load–deformation curves from the numerical models. The deformation *u* represents the vertical deformation of the rung end in the numerical model.

**Figure 27:**
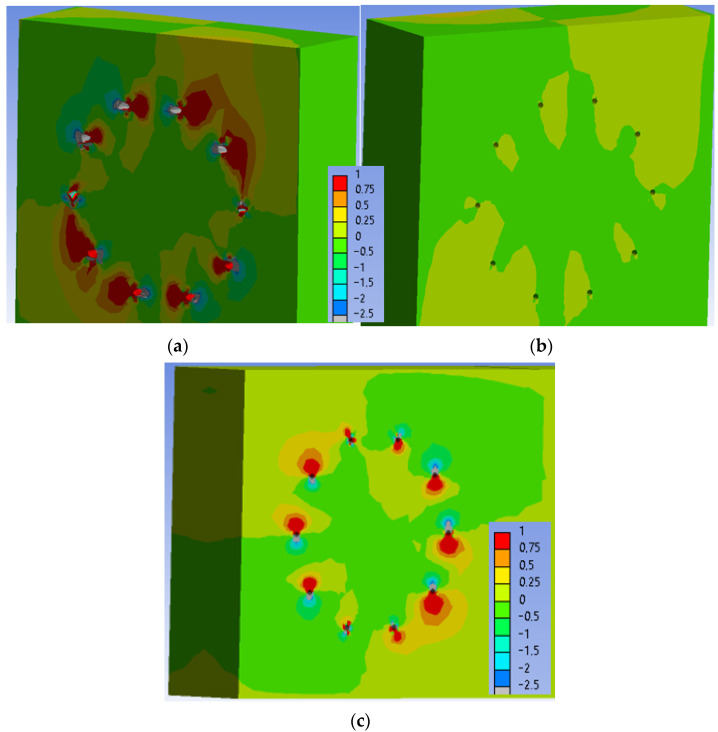
Tensile stress perpendicular to the grain [MPa] at 60% ULS, Experiment B: (**a**) inner side of the stand; (**b**) outer side of the stand; (**c**) rung.

Figure 27 shows the normal stress perpendicular to the grain in the individual elements of the connection at a load level of 60% ULS.

**Figure 28:**
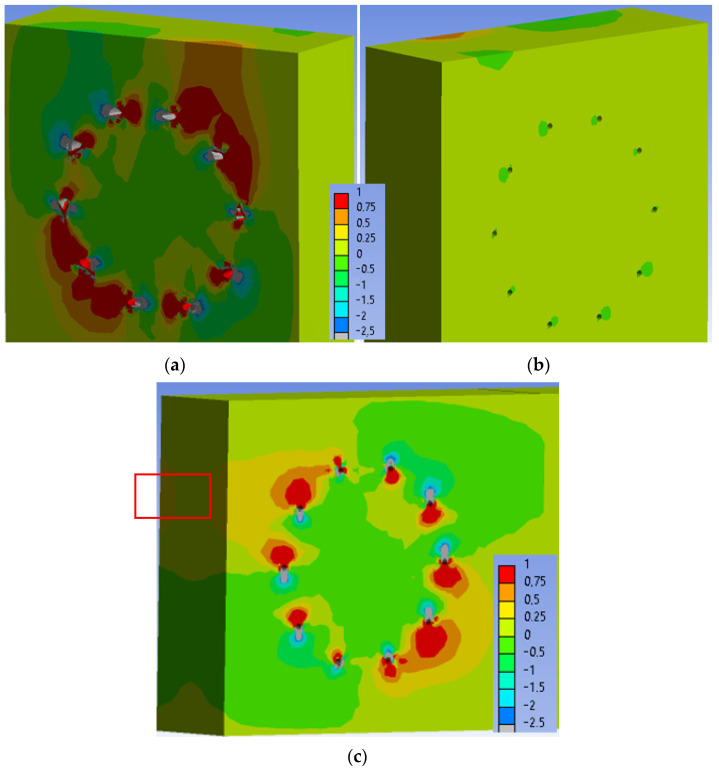
Tensile stress perpendicular to the grain [MPa] at 80% ULS, Experiment B: (**a**) inner side of the stand; (**b**) outer side of the stand; (**c**) rung.

Figure 28 shows the normal stress perpendicular to the grain in the individual elements of the connection at a load level of 80% ULS. The maximum tensile stress perpendicular to the grain (1 MPa) is also concentrated mainly near the holes at this load level. The stress in the critical area of the connection (marked in Figure 28c) increased to values of 0.25–0.50 MPa in very small range.

**Figure 29:**
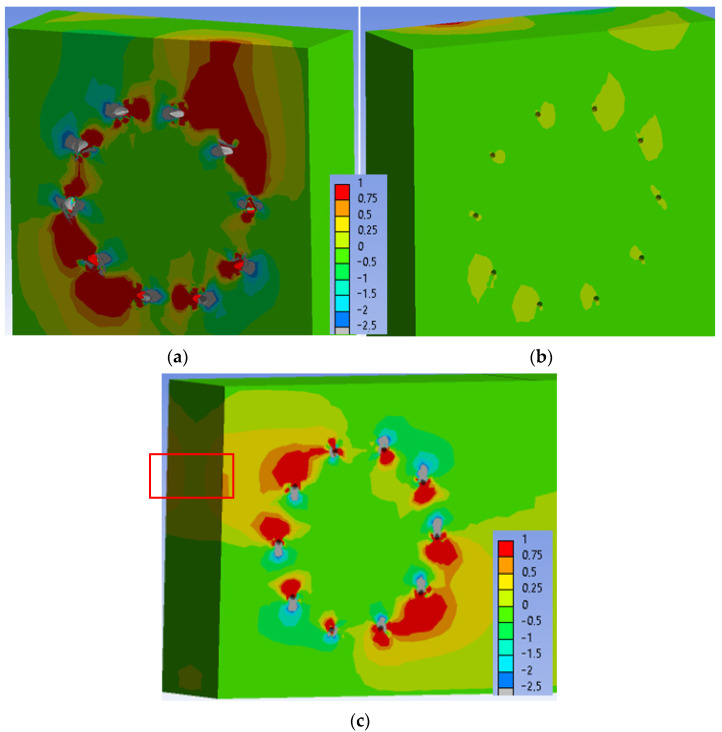
Tensile stress perpendicular to the grain [MPa] at 100% ULS, Experiment B: (**a**) inner side of the stand; (**b**) outer side of the stand; (**c**) rung.

Figure 29 shows the normal stress perpendicular to the grain in the individual elements of the connection at a load level of 100% ULS. Areas of the maximum tensile stress perpendicular to the grain (1 MPa) continued to expand around the holes at this load level. The stress in the critical area of the connection (marked in Figure 29c) remained unchanged with values of 0.25–0.50 MPa. The area of this stress did not increase significantly.

The results of the individual numerical models are listed in Table 15, Table 16 and Table 17. The first column indicates the load level. The second column shows the maximum force achieved during loading. The third column shows the corresponding value of the bending moment. The fourth column shows the actual deformation caused by the rotational stiffness of the semirigid connection. The fifth column contains the calculated value of the rotational stiffness based on the numerical model. The sixth and the seventh columns contain the standard deviation value and the average value of the experimental data. The eighth column contains the calculated rotational stiffness according to Eurocode 5 [10]. The ninth column shows the ratio between the rotational stiffness obtained from the numerical analysis and the rotational stiffness calculated according to Eurocode 5 [10].

## 4. Discussion

When testing the specimens by nondestructive quasi-static cyclic loading, the audible signals of the specimen failure did not occur, due to the low load level. This also applies to the 100% of the ultimate limit state. Each specimen was loaded with a value of about 2 kN before the start of the cyclic testing, in order to eliminate the concave shape of load–deformation curves. The fasteners were activated, and the initial consolidation of the connection and the initial slip took place by loading with this small force.

**Figure 30:**
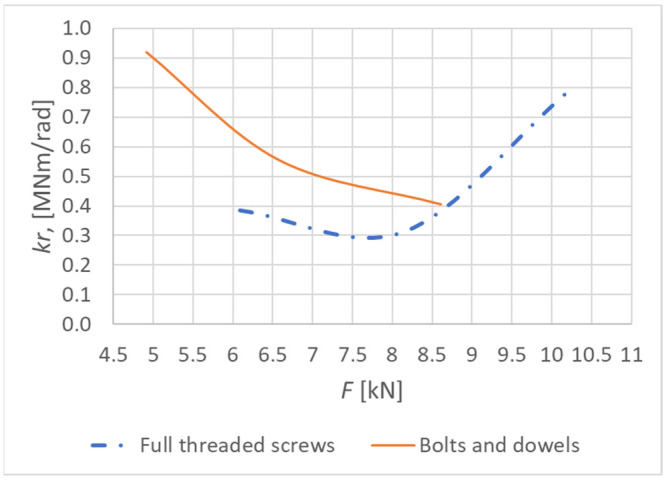
Comparison of the rotational stiffness trends.

An interesting course shown in Figure 30 can be observed by comparing the trends of rotational stiffness with the increasing load. The connection using bolts and dowels loses its rotational stiffness with the increasing load. On the contrary, the rotational stiffness of the connection using screws increases with the increasing load. The increase in rotational stiffness can be attributed to the axial forces that are generated in fasteners during its transverse loading. The transverse loading leads to the deformation of fasteners and thus to the loss of rotational stiffness in the initial phase of the loading. Increasing loading begins to generate axial forces in screws [40]. The transverse deformation is straightened again, thanks to the thread along the entire screw length. This phenomenon results in a gradual increase in the rotational stiffness during loading. Such a phenomenon does not occur with the connection using bolts and dowels, because the number of bolts is low and the effect of the washer and nut is not sufficient.

The results according to Eurocode 5 [9] represent a method for calculation of slip modulus. Possible variations of the results were created for comparison in order to capture the real rotational stiffness value using an analytical equation as accurately as possible. The rotational stiffness value from the last cycle (i.e., the fifth cycle) is used in the following tables.

Table 18 shows results obtained using the individual approaches. The third column represents the rotational stiffness value obtained experimentally. The fourth column contains the value obtained by the numerical modeling. The rotational stiffness value calculated according to Eurocode 5 [10] using the outer fastener diameter and the standard value of timber density is shown in the fifth column. The sixth column contains the values calculated according to Eurocode 5 [10] using the inner fastener diameter and the standard value of timber density. The rotational stiffness value calculated according to Eurocode 5 [10] using the actual measured timber density and diameter of the fastener shank is shown in the seventh column.

It can be seen in the table that all values (with the exception of screws to a load level of 80% ULS) are above the value of rotational stiffness calculated according to the standard equation for the slip modulus. From the point of view of a safer design, it is necessary to take into account the actual inner diameter, because it has a huge influence on the calculated values. The standard states the use of the fastener diameter, but does not define whether its actual inner core diameter or outer thread diameter. Based on the presented results, it is desirable to recommend the use of the inner core diameter of the fastener.

It is also possible to see relatively good accuracy with the numerical modeling. The numerical models thus provide details that are difficult to achieve during experimental testing, e.g., the stress curves during loading. No elements of connections were broken during experimental testing. The numerical models also correctly assumed this fact.

## 5. Conclusions

The paper was focused on the issue of the semirigid connection, which was composed of a timber rung and two stands using dowel-type fasteners. Six specimens were tested by quasi-static nondestructive cyclic loading. Three specimens were made from a combination of bolts and dowels and three specimens from fully threaded screws. The work required the creation of analytical assumptions, which were the basis for the design of the experimental testing and were subsequently used to compare the results.

Experimental testing proved that the connection created from a combination of bolts and dowels (Experiment A) is safe and reliable until the ultimate limit state. The rotational stiffness of this connection is higher than the standard estimate for the ultimate limit state during the entire loading process.

Testing further showed, that the connection created from fully threaded screws (Experiment B) was also safe and reliable until the ultimate limit state. The rotational stiffness of this connection was not higher than the standard estimate for the ultimate limit state for a load level corresponding to 80% ULS. The experiment indicates that in order to achieve more accurate results of rotational stiffness according to Eurocode 5 [9], it is necessary to use the inner core diameter of the fastener shank for the calculation of the slip modulus. Alternatively, it is possible to use the diameter of the fastener shank and the actual measured timber density. However, from a practical point of view, it is almost impossible to use the actual measured timber density for the standard calculations. The obtained data showed that the use of the inner core diameter of the fastener and the standard value of timber density for the standard calculations is sufficiently accurate.

The issue of determining the load-carrying capacity of connections in timber structures according to the European standards for the design of timber structures Eurocode 5 [10] is still under development. The proposed experiments should also contribute to this trend. The experiments were focused on determining the rotational stiffness of a semirigid connection of a rung and two stands using dowel-type mechanical fasteners.

The data from the experiments can be used for the practical design of this type of semirigid connection from the point of view of the rotational stiffness.

## Figures and Tables

**Table 1:** The course of the 60% ULS experiment setup.

Loading Step	Bolts and Dowels		Fully Threaded Screws	
	From	To	From	To
	(kN)	(kN)	(kN)	(kN)
Step 1	0	4.93	0	6.31
Step 2	Hold		Hold	
Step 3	4.93	0.85	6.31	1.05
Step 4	Hold		Hold	
Step 5	0.85	4.93	1.05	6.31
Step 6	Hold		Hold	
Repeating steps 3, 4, 5 four times.				

**Table 2:** Elastic constants of the material model for timber.

Timber Properties	Value	Unit
Young’s modulus in *X*	9200	MPa
Young’s modulus in *Y*	740	MPa
Young’s modulus in *Z*	400	MPa
Poisson’s ratio in *XY*	0.47	-
Poisson’s ratio in *YZ*	0.25	-
Poisson’s ratio in *XZ*	0.37	-
Shear modulus in *XY*	650	MPa
Shear modulus in *YZ*	38	MPa
Shear modulus in *XZ*	700	MPa

**Table 3:** Elastic constants of the material model of fasteners.

Steel Properties	Value	Unit
Young’s modulus	190,000	MPa
Poisson’s ratio	0.30	-

**Table 4:** Values for the plastic behavior of the material model for timber.

Hill Yield Criterion	Value	Unit
Yield strength in *X*	32	MPa
Yield strength in *Y*	1	MPa
Yield strength in *Z*	1	MPa
Yield strength in *XY*	6	MPa
Yield strength in *YZ*	3	MPa
Yield strength in *XZ*	6	MPa

**Table 5:** Values for the plastic behavior of the material model for steel.

**Bolts and Dowels**		
Yield strength	670	MPa
Ultimate strength	970	MPa
Hardening modulus	1000	MPa
**Fully Threaded Screws**		
Yield strength	690	MPa
Ultimate strength	1075	MPa
Hardening modulus	1000	MPa

**Table 6:** Results of quasi-static cyclic testing for Experiment A at 60% ULS.

Load Level	*F*_60% ULS_ [kN]	*M*_60% ULS_ [kNm]	*u* [mm]	*k*_r, 60% ULS_ [MNm/rad]	SD_,kr,60_ [MNm/rad]	AVG_,kr,60_ [MNm/rad]	*k*_r, u, EC5_ [MNm/rad]	Test/*u* [-]
60% ULS	4.93	3.80	3.06	0.955	0.023	0.917	0.323	2.96
			3.13	0.934				2.89
			3.21	0.911				2.82
			3.25	0.899				2.78
			3.27	0.894				2.77

**Table 7:** Results of quasi-static cyclic testing for Experiment A at 80% ULS.

Load Level	*F*_80% ULS_ [kN]	*M*_80% ULS_ [kNm]	*u* [mm]	*k*_r, 80% ULS_ [MNm/rad]	SD_,kr,80_ [MNm/rad]	AVG_,kr,80_ [MNm/rad]	*k*_r, u, EC5_ [MNm/rad]	Test/*u* [-]
80% ULS	6.50	5.01	6.85	0.563	0.005	0.554	0.323	1.74
			6.92	0.558				1.73
			6.98	0.553				1.71
			7.02	0.550				1.70
			7.04	0.548				1.70

**Table 8:** Results of quasi-static cyclic testing for Experiment A at 100% ULS.

Load Level	*F*_100% ULS_ [kN]	*M*_100% ULS_ [kNm]	*u* [mm]	*k*_r, 100% ULS_ [MNm/rad]	SD_,kr,100_ [MNm/rad]	AVG_,kr,100_ [MNm/rad]	*k*_r, u, EC5_ [MNm/rad]	Test/*u* [-]
100% ULS	8.66	6.67	12.58	0.408	0.002	0.401	0.323	1.26
			12.63	0.406				1.26
			12.68	0.404				1.25
			12.69	0.404				1.25
			12.70	0.404				1.25

**Table 9:** Results of quasi-static cyclic testing for Experiment B at 60% ULS.

Load Level	*F*_60% ULS_ [kN]	*M*_60% ULS_ [kNm]	*u* [mm]	*k*_r, 60% ULS_ [MNm/rad]	SD_,kr,60_ [MNm/rad]	AVG_,kr,60_ [MNm/rad]	*k*_r, u, EC5_ [MNm/rad]	Test/*u* [-]
60% ULS	6.31	4,86	9.39	0.383	0.002	0.386	0.323	1.19
			9.28	0.388				1.20
			9.34	0.385				1.19
			9.33	0.386				1.19
			9.33	0.386				1.19

**Table 10:** Results of quasi-static cyclic testing for Experiment B at 80% ULS.

Load Level	*F*_80% ULS_ [kN]	*M*_80% ULS_ [kNm]	*u* [mm]	*k*_r, 80% ULS_ [MNm/rad]	SD_,kr,80_ [MNm/rad]	AVG_,kr,80_ [MNm/rad]	*k*_r, u, EC5_ [MNm/rad]	Test/*u* [-]
80% ULS	8.10	6.24	15.28	0.314	0.000	0.313	0.323	0.97
			15.32	0.313				0.97
			15.33	0.313				0.97
			15.35	0.313				0.97
			15.36	0.313				0.97

**Table 11:** Results of quasi-static cyclic testing for Experiment B at 100% ULS.

Load Level	*F*_100% ULS_ [kN]	*M*_100% ULS_ [kNm]	*u* [mm]	*k*_r, 100% ULS_ [MNm/rad]	SD_,kr,100_ [MNm/rad]	AVG_,kr,100_ [MNm/rad]	*k*_r, u, EC5_ [MNm/rad]	Test/*u* [-]
100% ULS	10.17	7.83	8.77	0.688	0.069	0.784	0.323	2.13
			8.08	0.746				2.31
			7.67	0.787				2.44
			7.51	0.803				2.48
			6.73	0.896				2.77

**Table 12:** Results of quasi-static cyclic numerical modeling for Experiment A at 60% ULS.

Load Level	*F*_60%,num_ [kN]	*M*_60%, num_ [kNm]	*u* [mm]	*k*_r, 60%, num_ [MNm/rad]	SD_,kr,num,60_ [MNm/rad]	AVG_,kr,num,60_ [MNm/rad]	*k*_r, u, EC5_ [MNm/rad]	Num/u [-]
60% ULS	4.93	3.80	3.32	0.880	0.010	0.887	0.323	2.72
			3.32	0.880				2.72
			3.32	0.880				2.72
			3.25	0.889				2.75
			3.23	0.905				2.80

**Table 13:** Results of quasi-static cyclic numerical modeling for Experiment A at 80% ULS.

Load Level	*F*_80%, num_ [kN]	*M*_80%, num_ [kNm]	*u* [mm]	*k*_r, 80%, num_ [MNm/rad]	SD_,kr,num,80_ [MNm/rad]	AVG_,kr,num,80_ [MNm/rad]	*k*_r, u, EC5_ [MNm/rad]	Num/u [-]
80% ULS	6.50	5.01	6.71	0.575	0.022	0.609	0.323	1.72
			6.51	0.593				1.84
			6.32	0.611				1.89
			6.12	0.631				1.95
			6.09	0.634				1.96

**Table 14:** Results of quasi-static cyclic numerical modeling for Experiment A at 100% ULS.

Load Level	*F*_100%, num_ [kN]	*M*_100%, num_ [kNm]	*u* [mm]	*k*_r, 100%, num_ [MNm/rad]	SD_,kr,num,100_ [MNm/rad]	AVG_,kr,num,100_ [MNm/rad]	*k*_r, u, EC5_ [MNm/rad]	Num/u [-]
100% ULS	8.66	6.67	14.67	0.350	0.013	0.370	0.323	1.08
			14.25	0.360				1.11
			13.84	0.371				1.15
			13.43	0.382				1.18
			13.32	0.385				1.19

**Table 15:** Results of quasi-static cyclic numerical modeling for Experiment B at 60% ULS.

Load Level	*F*_60%,num_ [kN]	*M*_60%, num_ [kNm]	*u* [mm]	*k*_r, 60%, num_ [MNm/rad]	SD_,kr,num,60_ [MNm/rad]	AVG_,kr,num,60_ [MNm/rad]	*k*_r, u, EC5_ [MNm/rad]	Num/u [-]
60% ULS	6.31	4.86	7.68	0.469	0.007	0.457	0.323	1.45
			7.81	0.461				1.43
			7.92	0.454				1.41
			8.02	0.449				1.39
			7.92	0.454				1.41

**Table 16:** Results of quasi-static cyclic numerical modeling for Experiment B at 80% ULS.

Load Level	*F*_80%, num_ [kN]	*M*_80%, num_ [kNm]	*u* [mm]	*k*_r, 80%, num_ [MNm/rad]	SD_,kr,num,80_ [MNm/rad]	AVG_,kr,num,80_ [MNm/rad]	*k*_r, u, EC5_ [MNm/rad]	Num/u [-]
80% ULS	6.31	6.24	17.09	0.281	0.024	0.304	0.323	0.87
			17.53	0.274				0.85
			15.92	0.302				0.93
			14.59	0.329				1.02
			14.39	0.334				1.03

**Table 17:** Results of quasi-static cyclic numerical modeling for Experiment B at 100% ULS.

Load Level	*F*_100%, num_ [kN]	*M*_100%, num_ [kNm]	*u* [mm]	*k*_r, 100%, num_ [MNm/rad]	SD_,kr,num,100_ [MNm/rad]	AVG_,kr,num,100_ [MNm/rad]	*k*_r, u, EC5_ [MNm/rad]	Num/u [-]
100% ULS	10.17	7.83	10.36	0.582	0.038	0.638	0.323	1.80
			9.85	0.612				1.89
			9.50	0.635				1.97
			8.97	0.672				2.08
			8.78	0.687				2.13

**Table 18:** Comparison of rotational stiffness results.

Type of Fastener	Test	*k*_r, test_ [MNm/rad]	*k*_r, num_ [MNm/rad]	*k*_r, EC5_ [MNm/rad]	*k*_r, RS_ [MNm/rad]	*k*_r, RSD_ [MNm/rad]
Bolts and dowels	60% ULS	0.894	0.905	0.323	0.293	0.299
	80% ULS	0.548	0.634			
	100% ULS	0.404	0.385			
Fully threaded screws	60% ULS	0.386	0.454		0.202	0.206
	80% ULS	0.313	0.334			
	100% ULS	0.896	0.687			

## Data Availability

Data are contained within the article.

## References

[1] Granholm H. (1949). Om Sammansatta Balkar och Pelare med Särskild Hänsyn Till Spikade Träkonstruktionr. Handlingar 88.

[2] Granholm H. (1963). Der Einsturz des Bogengerüstes der Sandöbrücke.

[3] Ehlbeck J. Load-carrying capacity and deformation characteristics of nailed joints. Proceedings of the CIB-W18, Paper 12-7-1.

[4] Dubas P., Gehri E., Steurer T. (1981). Einführung in die Norm SIA 164 (1981)–Holzbau.

[5] Lokaj A., Dobes P., Sucharda O. (2020). Effects of Loaded End Distance and Moisture Content on the Behavior of Bolted Connections in Squared and Round Timber Subjected to Tension Parallel to the Grain. Materials.

[6] Ehlbeck J., Werner H. (1988). Untersuchungen über die Tragfähigkeit von Stabdübelverbindungen. Holz Roh Werkst..

[7] Stoy W. (1942). Holz-Nagelbau nach DIN 1052, 3. Ausgabe, 1940. 4. Ergänzte und Verbesserte Auflage.

[8] Ehlbeck J., Larsen H.J., Barnes M., Brauner A., Galligan W., Leichti R., Soltis L. (1993). Eurocode 5 design of timber structures: Joints. International Workshop on Wood Connectors.

[9] Jorissen A. (1998). Double Shear Timber Connections with Dowel Type Fasteners. Ph.D. Thesis.

[10] (2006). Eurocode 5: Design of Timber Structures—Part 1-1: General—Common Rules and Rules for Buildings.

[11] Kozelouh B. (1998). Timber Structures According to Eurocode 5; STEP 1: Design and Construction Materials; Translated by Bohumil Kozelouh.

[12] Solarino F., Giresini L., Chang W.-S., Huang H. (2017). Experimental Tests on a Dowel-Type Timber Connection and Validation of Numerical Models. Buildings.

[13] Vavrusova K., Mikolasek D., Lokaj A., Klajmonova K., Sucharda O. (2016). Determination of carrying capacity of steel-timber joints with steel rods glued-in parallel to grain. Wood Res..

[14] Vassiliou V., Barboutis I., Kamperidou V. (2016). Strength of Corner and Middle Joints of Upholstered Furniture Frames Constructed with Black Locust and Beech Wood. Wood Res..

[15] Cai Y., Young B. (2019). Effects of end distance on thin sheet steel bolted connections. Eng. Struct..

[16] Požgaj A., Kürjatko S. (1986). Wood properties of spruce from forests affected by pollution in Czechoslovakia. IAWA J..

[17] Mirski R., Dziurka D., Chuda-Kowalska M., Wieruszewski M., Kawalerczyk J., Trociński A. (2019). The Usefulness of Pine Timber (*Pinus sylvestris* L.) for the Production of Structural Elements. Part I: Evaluation of the Quality of the Pine Timber in the Bending Test. Materials.

[18] Mirski R., Dziurka D., Chuda-Kowalska M., Kawalerczyk J., Kuliński M., Łabęda K. (2020). The Usefulness of Pine Timber (*Pinus sylvestris* L.) for the Production of Structural Elements. Part II: Strength Properties of Glued Laminated Timber. Materials.

[19] Nowak T., Karolak A., Sobótka M., Wyjadłowski M. (2019). Assessment of the Condition of Wharf Timber Sheet Wall Material by Means of Selected Non-Destructive Methods. Materials.

[20] Nowak T., Patalas F., Karolak A. (2021). Estimating Mechanical Properties of Wood in Existing Structures—Selected Aspects. Materials.

[21] Bragov A., Igumnov L., dell’Isola F., Konstantinov A., Lomunov A., Iuzhina T. (2020). Dynamic Testing of Lime-Tree (Tilia Eu-ropoea) and Pine (Pinaceae) for Wood Model Identification. Materials.

[22] Olaoye K., Aguda L., Ogunleye B. (2021). Prediction of Mechanical Properties of Hardwood Species Using the Longitudinal Vib-ration Acoustic Method. For. Prod. J..

[23] Martínez R.D., Balmori J.-A., Llana D.F., Bobadilla I. (2020). Wood Density and Moisture Content Estimation by Drilling Chips Extraction Technique. Materials.

[24] Dobes P., Lokaj A., Mikolasek D. (2022). Load-Carrying Capacity of Double-Shear Bolted Connections with Slotted-in Steel Plates in Squared and Round Timber Based on the Experimental Testing, European Yield Model, and Linear Elastic Fracture Mechanics. Materials.

[25] Braun M., Pełczyński J., Al Sabouni-Zawadzka A., Kromoser B. (2022). Calibration and Validation of a Linear-Elastic Numerical Model for Timber Step Joints Based on the Results of Experimental Investigations. Materials.

[26] Burawska-Kupniewska I., Beer P. (2021). Near-Surface Mounted Reinforcement of Sawn Timber Beams-FEM Approach. Materials.

[27] Zhou S., Li Z., Feng S., Zhu H., Kang S. (2021). Effects of bolted connections on behaviour of timber frames under combined vertical and lateral loads. Constr. Build. Mater..

[28] Wang M., Song X., Gu X., Tang J. (2019). Bolted glulam beam-column connections under different combinations of shear and bending. Eng. Struct..

[29] Wang X.T., Zhu E.C., Niu S., Wang H.J. (2021). Analysis and test of stiffness of bolted connections in timber structures. Constr. Build. Mater..

[30] Johanides M., Kubíncová L., Mikolášek D., Lokaj A., Sucharda O., Mynarčík P. (2021). Analysis of Rotational Stiffness of the Timber Frame Connection. Sustainability.

[31] Johanides M., Mikolasek D., Lokaj A., Mynarcik P., Marcalikova Z., Sucharda O. (2021). Rotational Stiffness and Carrying Capacity of Timber Frame Corners with Dowel Type Connections. Materials.

[32] Johanides M., Lokaj A., Mikolasek D., Mynarcik P., Dobes P., Sucharda O. (2022). Timber Semirigid Frame Connection with Improved Deformation Capacity and Ductility. Buildings.

[33] LaborTech https://www.labortech.cz.

[34] Ahlborn. https://www.ahlborn.com.

[35] (1994). Timber Structures. Joints Made with Mechanical Fasteners. General Principles for the Determination of Strength and Deformation Characteristics. Czech Office for Standards.

[36] Ansys. https://www.ansys.com/.

[37] Brožovský, Jiří a Alois Materna (2012). Metoda Konečných Prvkůve Stavební Mechanice. https://docplayer.cz/414068-Metoda-konecnych-prvku-ve-stavebni-mechanice.html.

[38] David M. (2012). Numerické Modelování Vybraných Spojů Dřevěných Konstrukcí.

[39] Gunderson R.A., Goodman J.R., Bodig J. (1973). Plate Tests for Determination of Elastic Parameters of Wood. Wood Sci..

[40] Larsen H.J., Reestrup V. (1969). Tests on screws in wood. Bygn. Medd..

